# Assessment of Bacterial *bph* Gene in Amazonian Dark Earth and Their Adjacent Soils

**DOI:** 10.1371/journal.pone.0099597

**Published:** 2014-06-13

**Authors:** Maria Julia de Lima Brossi, Lucas William Mendes, Mariana Gomes Germano, Amanda Barbosa Lima, Siu Mui Tsai

**Affiliations:** 1 Cellular and Molecular Biology Laboratory, Center for Nuclear Energy in Agriculture, University of São Paulo, Piracicaba, SP, Brazil; 2 Brazilian Agricultural Research Corporation, Embrapa Soybean, Londrina, PR, Brazil; University of Hyderabad, India

## Abstract

Amazonian Anthrosols are known to harbour distinct and highly diverse microbial communities. As most of the current assessments of these communities are based on taxonomic profiles, the functional gene structure of these communities, such as those responsible for key steps in the carbon cycle, mostly remain elusive. To gain insights into the diversity of catabolic genes involved in the degradation of hydrocarbons in anthropogenic horizons, we analysed the bacterial *bph* gene community structure, composition and abundance using T-RFLP, 454-pyrosequencing and quantitative PCR essays, respectively. Soil samples were collected in two Brazilian Amazon Dark Earth (ADE) sites and at their corresponding non-anthropogenic adjacent soils (ADJ), under two different land use systems, secondary forest (SF) and manioc cultivation (M). Redundancy analysis of T-RFLP data revealed differences in *bph* gene structure according to both soil type and land use. Chemical properties of ADE soils, such as high organic carbon and organic matter, as well as effective cation exchange capacity and pH, were significantly correlated with the structure of *bph* communities. Also, the taxonomic affiliation of *bph* gene sequences revealed the segregation of community composition according to the soil type. Sequences at ADE sites were mostly affiliated to aromatic hydrocarbon degraders belonging to the genera *Streptomyces*, *Sphingomonas*, *Rhodococcus*, *Mycobacterium*, *Conexibacter* and *Burkholderia*. In both land use sites, shannon's diversity indices based on the *bph* gene data were higher in ADE than ADJ soils. Collectively, our findings provide evidence that specific properties in ADE soils shape the structure and composition of *bph* communities. These results provide a basis for further investigations focusing on the bio-exploration of novel enzymes with potential use in the biotechnology/biodegradation industry.

## Introduction

Amazonian Dark Earth (ADE), locally termed *‘Terra Preta de Índio’*, are anthropogenic soil horizons built-up by the Pre-Colombian Indians between 500 and 8,700 years ago. These soil sites were formed by the progressive deposit of materials and organic compounds, such as charcoal, bone, and pottery sheds, which gradually shifted the natural physical and chemical properties of the soil. As a result, relatively infertile Amazon soils were progressively converted into highly fertile spots through processes like increasing the cation exchange capacity and the nutrient content, as well as promoting the stabilization of the soil physical structure [1], [2]. Substantial increments of organic material in these sites gradually increased the carbon content, yielding to the formation of soil spots with a high proportion of incompletely combusted biomass (biochar). These spots have been reported to reach up to a 70-fold higher amount of carbon than native soils at adjacent locations (ADJ) [3].

The existence of ADE sites close to their natural ADJ soil locations, which present the same geological history, provides a unique opportunity to investigate the role of biotic and abiotic factors influencing the microbial community assembly and dynamics at these sites. Previous studies revealed that ADE and ADJ sites present differences in microbial community composition, and bacterial diversity has been reported to be higher at ADE sites [4], [5], [6], [7],[8]. Most of these studies rely on comparisons between the taxonomic profiles of these communities (i.e., based on the taxonomic bacterial 16S rRNA gene). In this sense, the extent to which the local environment shapes the functional profiles of these communities, and influences their performance, remains mostly elusive [9], [10], [11], [12], [13].

Soil is one of the most biodiverse ecosystems on Earth, being able to support communities from multiple trophic levels, which are constantly performing the metabolism of diverse and complex substrates. The extreme spatial and temporal heterogeneity of the soil matrix, paired with the myriad of internal and external feedbacks, are known to determine the structure and function of these communities [14]. Microbes are involved in many ecosystem processes, including biodegradation, decomposition and mineralization, inorganic nutrient cycling, disease causation and suppression, and pollutant removal. Soil disturbances are known to cause shifts in microbial activities, shifting the rate of these processes and triggering impacts on the ecosystem performance [15]. Several environmental factors are known to affect microbial community composition in the soil, including soil temperature, moisture, texture, carbon content, nutrient availability, pH, land use history, seasonality, and the content of incompletely combusted biomass, such as biochar [13], [16], [17], [18], [19].

The biochar content is the major physical distinction between ADE and their ADJ soils, which is also known to play an important role in global carbon biogeochemistry [20]. In anthrosols, such as ADE, it is predicted that distinct microbial communities can perform unique processes, such as the retention of high-labile carbon [5]. Despite the unique and specialized capabilities of these soils, functional assessments of their microbial community, particularly those of genes encoding important steps in the carbon cycle, are still scarce. Biodegradation through bacterial activity is one of the most important processes occurring in soils regarding organic matter recycling. This process involves genes acting on key steps in the carbon cycle, for the turnover of more recalcitrant organic carbon, as well as for pollutant degradation in the ecosystem [21]. This process, along with biosynthesis, largely governs the carbon cycle in the environment, which is dependent on microbial enzymatic activities that most often use organic compounds as a primary energy source [22].

The primary step involved in the aerobic microbial degradation of aromatic hydrocarbons is an oxidative attack [23], [24], where enzymes named oxygenases are responsible for the insertion of molecular oxygen into aromatic benzene rings [25]. Genes encoding these enzymes have been characterized in *Rhodococcus*, *Acinetobacter*, *Pseudomonas*, *Mycobacterium*, *Burkhoderia*, *inter alia* [25], [26]. The α-subunit of oxigenases is known as the catalytic domain involved in the transfer of electrons to oxygen molecules. Due its DNA sequence conservation, this subunit has been currently used as a target gene for the detection of such enzymes in complex communities [21], [27], [28], [29].

In this study, we evaluated the structure, composition and abundance of the bacterial catabolic gene Biphenyl Dioxygenase (*bph*) involved in aromatic hydrocarbon degradation in Amazonian Dark Earth and their adjacent soil locations. We aimed to determine the role of anthropogenic action in the diversity of the *bph* gene in soil bacterial communities. Understanding the diversity of specific bacterial genes in ADE should led to future studies that investigate the microbial ecology of anthropogenic altered soils, especially in regards to their potential source of novel enzymes. Collectively, this study characterized this catabolic gene occurring in Amazonian Dark Earth, and compared the profiles with those obtained from adjacent sites, as well as under distinct land use systems.

## Materials and Methods

### Ethic statement

No specific permits were required for the described field studies. The locations are not protected. The field studies did not involve endangered or protected species.

### Study sites, sample collection and soil chemical analyses

Studied sites are located at the Caldeirão Experimental Station of Amazon Brazilian Agricultural Research Corporation (Embrapa) in Iranduba County in the Brazilian Central Amazon (03°26′00" S, 60°23′00" W). A detailed description of the soil sampling locations is given by Taketani et al [8]. Briefly, the four sites sampled are composed of two Amazonian Dark Earth (ADE) and their two correspondent adjacent soils (Haplic Acrisol, ADJ). These sites are under a ∼35 year-old secondary forest (SF) or under manioc (*Manihot esculenta*) cultivation (M). Hereafter these sites are termed as ADE-SF, ADJ-SF, ADE-M and ADJ-M. Soil samples were taken in triplicate from the topsoil layer (ca. top 10 cm), and the overlaying litter was discarded. Each sample contained approximately 300 g of soil and was transported to the laboratory at 4°C to further processing (<24 h). A portion of the samples were frozen (−20°C) for total DNA extraction while the other portion was kept at 4°C for chemical measurements.

Chemical analyses were performed at Amazon Embrapa (Manaus, Brazil), according to instructions provided by the Embrapa protocol [30]. Briefly, soil samples were analysed in triplicate for pH (H_2_O, 1∶2.5); H+Al (calcium extractor 0.5 mol L^−1^, pH 7.0); sum of bases (SB); soil organic matter (SOM); soil organic carbon (SOC; Walkely-Black method); extractable fraction of Al, Ca, and Mg (1 M KCl); extractable fraction of P and K (double acid solution of 0.025 M sulphuric acid and 0.05 M hydrochloric acid Mehlich 1); and effective cation exchange capacity (eCEC).

### Total soil DNA extraction and PCR amplifications for T-RFLP

Total DNA was extracted using 0.25 g of soil as an initial material. Extractions were carried out in triplicate for each site, using the PowerSoil DNA isolation kit (MoBio Laboratories, Carlsbad, CA, USA), according to the manufacturer's protocol. DNA quality and quantity were measured spectrophotometrically using NanoDrop 1000 (Thermo Scientific, Waltham, EUA).

For T-RFLP analyses the bacterial 16S rRNA gene was amplified with the primer set 27F - FAM labelled (5′ AGA GTT TGA TCC TGG CTC AG 3′) and 1492r (5′ ACC TTG TTA CGA CTT 3′) [31]. The *bph* gene was amplified with the primer set BPHD F1 – FAM labelled (5′ TAY ATG GGB GAR GAY CCI GT 3′) and BPHD R0 (5′ ACC CAG TTY TCI CCR TCG TC 3′) [21]. For 16S rRNA gene amplification, PCR reactions were carried out in a volume of 25 µL containing 2.5 µL reaction buffer 10× (Invitrogen, Carslbad, CA, USA), 1.5 µL MgCl_2_ (50 mM), 1 µL of each primer (5 pmol µL^−1^), 0.2 µL (5 U) of Platinum Taq DNA polymerase (Invitrogen), 0.5 mL of deoxyribonucleotide triphosphate mixture (2.5 mM), 0.25 µL of bovine serum albumin (1 ng mL^−1^), 1 µL of DNA template (ca. 10 ng) and 18.05 µL of sterilized ultrapure water. Amplifications were performed in the GeneAmp PCR System 9700 thermal cycler (Applied Biosystems, Foster City, CA, USA). Reaction conditions were 94°C for 3 min, followed by 35 cycles of 94°C for 30 s, 59°C for 45 s, and 72°C for 1 min with a final extension step at 72°C for 15 min. For the *bph* gene amplification, PCR reactions were carried out in a volume of 25 µL containing 2.5 µL reaction buffer 10× (Invitrogen, Carslbad, CA, USA), 1.5 µL MgCl_2_ (50 mM), 1.25 µL of each primer (5 pmol µL^−1^), 0.5 µL (5 U) of Platinum Taq DNA polymerase (Invitrogen), 0.3 µL of deoxyribonucleotide triphosphate mixture (2.5 mM), 1 µL of DNA template (ca. 10 ng) and 16.7 µL of sterilized ultrapure water. For *bph* gene, similar PCR cycling conditions were used, except the annealing temperature that was set at 60°C. Negative PCR controls (without DNA template) and positive controls (using the *Escherichia coli* ATCC 25922 DNA for the 16S rRNA gene and the DSM 6899 *Pseudomonas putida* DNA for *bph*) were run in parallel for both amplifications. After the amplifications, 5 µL of obtained products (ca. 60 ng) was digested with the endonuclease *HhaI* (Invitrogen) in 15 µL reaction for 3 h at 37°C. Obtained fragments were further purified using sodium acetate/EDTA precipitation and then mixed with 0.25 µL of the Genescan 500 ROX size standard (Applied Biosystems) and 9.75 µL of deionized formamide. Prior to fragment analysis, samples were denatured at 95°C for 5 min and chilled on ice. Analysis of terminal restriction fragment (T-RF) sizes and quantities was performed on an ABI PRISM 3100 genetic analyzer (Applied Biosystems).

T-RFLP profiles were analysed using PeakScanner v1.0 software (Applied Biosystems, Foster City, CA, USA). Terminal restriction fragments (T-RFs) of less than 25 bp were excluded prior to the analysis. The total values of T-RFs for each soil sample were pulled together to construct a Venn's diagram showing shared T-RFs among samples. The relative abundance of a single T-RF was calculated as percent fluorescence intensity relative to total fluorescence intensity of the peaks [32]. Data from individual samples were combined to soil chemical parameters and subjected to multivariate analysis using Canoco 4.5 (Biometris, Wageningen,The Netherlands) and Primer6 (PrimerE, Ivybridge, United Kingdom). All matrices were initially analysed using de-trended correspondence analysis (DCA) to evaluate the length of the gradient of the species distribution; this analysis indicated linearly distributed data (length of gradient <3), revealing that the best-fit mathematical model for the data was the redundancy analysis (RDA). Forward selection (FS) and the Monte Carlo permutation test were applied with 1,000 random permutations to verify the significance of soil chemical properties upon the microbial community. In addition to *P* values for the significance of each soil chemical property, RDA and Monte Carlo permutation test supplied information about the marginal effects of environmental variables, quantifying the amount of variance explained by each factor. We used ANOSIM based on relative abundance of T-RFs to test for statistical differences between samples.

### 454-Pyrosequencing analyses of the bacterial *bph* gene

A partial region of the *bph* gene was amplified for the 454-pyrosequencing using the primer set BPHD F3 (5′ ACT GGA ART TYG CIG CVG A 3′) and BPHD R0 (5′ ACC CAG TTY TCI CCR TCG TC 3′) [20] containing specific Roche 454-pyrosequencing adaptors and barcodes of 8 bp. The expected fragment size was ca. 520 bp. Three independent amplifications were performed for each sample. The 20 µL PCR mixture contained 1× FastStart High Fidelity Reaction Buffer (Roche Diagnostics, Basel, Switzerland), 1.25 mM of each primer, 150 ng mL^−1^ of bovine serum albumin (New England BioLabs, Ipswich, MA, USA), 0.2 mM of dNTPs, 0.5 mL (2.5 U) of FastStart High Fidelity PCR System Enzyme Blend (Roche Diagnostics) and 4 ng of template DNA. The PCR conditions were optimized using the genomic DNA of *Burkholderia xenovorans* LB400 [33], which carries one of the target dioxygenase genes. Amplifications were performed as follows: 95°C for 3 min, 30 cycles of 95°C for 45 s, 60°C for 45 s and 72°C for 40 s, with the final extension of 72°C for 4 min. Triplicate PCR products containing the expected fragment size were purified using the QIAquick Gel Extraction Kit (Qiagen, Hilden, Germany) and QIAquick PCR Purification Kit (Qiagen). DNA concentrations were determined using the NanoDrop ND-1000 spectrophotometer (NanoDrop Technologies, Wilmington, DE, USA). Purified PCR products were pooled and subjected to pyrosequencing using the FLX sequencing system (454 Life Sciences, Branford, CT, USA).

Raw data was filtered for valid sequences using the FunGene Pipeline Repository (http://fungene.cme.msu.edu/FunGenePipeline/). Quality sequences were translated in the correct frame of aminoacids using the RDP FrameBot tool. The RDP pipeline extracted a set of representative sequences from known *bph* sequences to use as subject sequences with FrameBot. The FrameBot produces an optimal alignment between the query and the subject sequences in the presence of frameshifts. Only the protein pairwise alignment with the best score was reported. Protein sequences passing FrameBot were aligned with HMMER using a model trained on the same set of representative sequences used by FrameBot. The aligned protein sequences were chopped at position 351 of the reference sequence of *Pseudomonas putida* F1 (YP_001268196). The total valid sequences were rarefied to the smallest number of sequences per sample in order to minimize effects of sampling effort upon analysis.

Distance matrices were constructed using the MOTHUR software [34]. The resulting matrices were used to estimate the number of operational protein families (OPF) (i.e. group of proteins that share a common evolutionary origin) and to estimate richness (i.e. Chao1, Jackknife, and ACE indices) and diversity (i.e. Shannon and Simpson indices). Rarefaction curves were constructed at a cutoff level of 94% of amino acid identity. MOTHUR was also used to perform ∫-Libshuff comparisons between the four studied sites. The Good's coverage estimator was used to calculate the sample coverage using the formula C =  1-(n_i_/N), where *N* is the total number of sequences analysed and *n_i_* is the number of reads that occurs only once among the total number of reads analysed at a cutoff value of 94% of amino acid identity [35]. Unweighted UniFrac distances among communities were estimated using a tree constructed *de novo* using FastTree. One representative sequence per OPF was selected and subjected to taxonomic affiliation by the comparison tool of NCBI Tblastx (GenBank) using Blast2Go [36].

Sequence data generated by 454-pyrosequencing are available at the MG-RAST server (http://metagenomics.anl.gov) under the project ‘Diversity of bhp gene in Amazon soils' (ID 8489) and accession numbers 4557319.3 (ADE_SF), 4557318.3 (ADE_M), 4557321.3 (ADJ_SF) and 4557320.3 (ADJ_M).

### Quantitative PCR (qPCR) of the bacterial 16S rRNA and *bph* genes

The bacterial 16S rRNA gene was amplified with the primer set U968F (5' AAC GCG AAG AAC CTT AC 3 ') and R1387 (5' CGG TGT GTA CAA GGC CCG GGA ACG 3 ') [37], which amplify a fragment of approximately 400 bp. The 520 bp fragment of the *bph* gene was amplified with the primer set BPHD F3 (5′ ACT GGA ART TYG CIG CVG A 3′) and BPHD R0 (5′ ACC CAG TTY TCI CCR TCG TC 3′) [21]. qPCR reactions were performed in 10 µL containing 5 µL of SYBR green PCR master mix (Fermentas, Brazil), 1 µL of each primer (5 pmol µL^−1^), 1 µL of DNA template (ca. 10 ng) and 2 µL of sterilized ultrapure water. Thermocycling conditions for the 16S rRNA gene were set as follows: 94°C for 10 min; 40 cycles of 94°C for 30 s, 56°C for 30 s and 72°C for 40 s. Amplification specificity was checked by a melting curve and the data collection was performed at every 0.7°C. qPCR reactions for the *bph* gene were performed at similar conditions, except for the annealing temperature set at 60°C. Reactions were performed in a StepOnePlus system (Applied Biosytems). The Cts values (cycle threshold) were used as standers for determining the amount of DNA template in each sample. Standard curves were produced for the 16S rRNA and *bph* genes using specific cloned fragments. Gene fragments were quantified in a spectrophotometer (190 a 840 nm - NanoDrop ND-1000) and diluted (10^7^ to 10^3^ genes µL^−1^ for the 16S rRNA gene and 10^6^ to 10^2^ genes µL^−1^ for *bph*) to generate each specific standard curves. The gene copy numbers in different soil samples were expressed as log copy numbers of the gene per gram of soil. Statistical comparisons were performed using one-way ANOVA (Tukey's test).

## Results

### Variation in soil chemical properties

Soil chemical properties were measured for each individual sample collected in ADE and ADJ sites (for a detailed description see Table S1). Statistical differences were observed using Tukey's test. Overall, soil chemical properties of ADE-SF were chemically similar to ADE-M. Likewise, ADJ-SF chemical properties were also very similar to ADJ-M. As expected, major differences were attributed mostly to soil type rather than the land use history.

Higher soil pH values were observed in ADE rather than ADJ sites. While ADJ soils were very acidic with a pH of 3.53 (ADJ-SF) and 3.74 (ADJ-M), ADE sites were only weakly acidic with a pH of 5.51 (ADE-SF) and 5.41 (ADE-M). Sites at ADE showed lower total and exchangeable Al (H+Al), a phenomenon that is likely directly connected to observed variations in soil pH.

Soil organic carbon (SOC), soil organic matter (SOM) and effective cation exchange capacity (eCEC) were higher in ADE sites. Different land uses did not influence these properties in ADJ soils; however, the same properties showed significantly higher values in the site under secondary forest rather than in manioc cultivation, in ADE soil locations. In detail, SOC, SOM and eCEC values were approximately 30% higher in the ADE-SF site when compared to the ADE-M.

### Assessment of community structures based on the bacterial 16S rRNA and *bph* genes

T-RFLP analyses for the bacterial 16S rRNA and *bph* genes were performed for the four sites. The obtained profiles were used to determine the richness of terminal restriction fragments (T-RFs) and to perform the multivariate analyses. A total of 152, 144, 147 and 141 T-RFs were obtained for the analysis of the bacterial 16S rRNA gene in ADE-SF, ADE-M, ADJ-SF and ADJ-M sites, respectively. There were 14 T-RFs detected as dominant throughout all sites, accounting for >50% of the total fluorescence detected for the 16S rRNA gene analyses. Tukey's test (*P*>0.05) indicated no difference between sites in the richness of T-RFs for the obtained profiles of bacterial 16S rRNA gene.

For the *bph* gene analysis no dominant T-RFs were found, possibly due to the high heterogeneity of this gene. There were 90, 78, 73 and 69 T-RFs in ADE-SF, ADE-M, ADJ-SF and ADJ-M sites, respectively. Samples from ADE sites showed statistically higher richness of T-RFs (Tukey's test) than the observed at ADJ sites (*P*>0.05).

**Figure 1:**
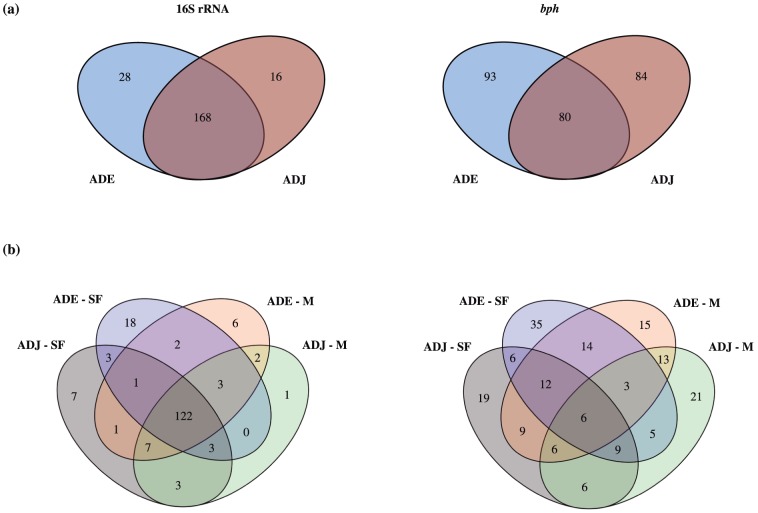
Venn's diagram of T-RFs for 16S rRNA and *bph* genes according to (a) soil types and (b) soil types and land uses. ADE  =  Amazon Dark Earth; ADJ  =  Adjacent soils; SF  =  Secondary Forest; M  =  Manioc cultivation.

The Venn's diagram according to soil type showed that ADE and ADJ soils shared more common T-RFs for the bacterial 16S rRNA rather than for *bph* gene. Also, the number of unique T-RFs was higher in ADE sites for both assessed genes (Figure 1a). Conversely, Venn's diagram combining the four sites showed a core containing 122 T-RFs for the bacterial 16S rRNA gene, while the distribution of T-RFs for the *bph* gene was more site specific, and only 6 T-RFs comprised a common core (Figure 1b).

**Figure 2:**
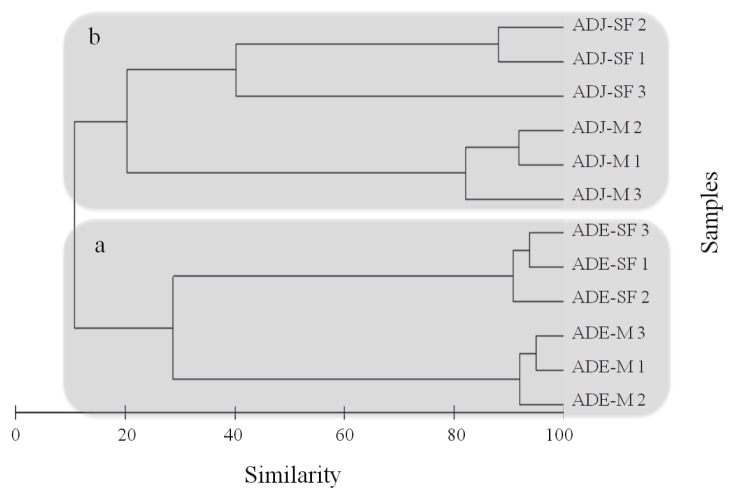
Clustering analysis of T-RFLP data based on Bray-Curtis similarity for the *bph* gene. ‘a’ and ‘b’ indicate the segregation patterns according to soil type. ADE  =  Amazon Dark Earth; ADJ  =  Adjacent soils; SF  =  Secondary Forest; M =  Manioc cultivation.

Clustering analysis of T-RFLP data for the *bph* gene segregated samples according to soil type and land use (Figure 2). This analysis revealed the formation of two main clusters: the first cluster (a) included samples from ADE soils (SF and M) and the second (b) samples from ADJ soils (SF and M). This analysis also revealed that ADE and ADJ sites segregated at 12% of similarity. Concerning the ADE sites, land use systems separated different land uses at 27% of similarity. Conversely, for ADJ sites, land use systems differed at 20% similarity (Figure 2).

**Figure 3:**
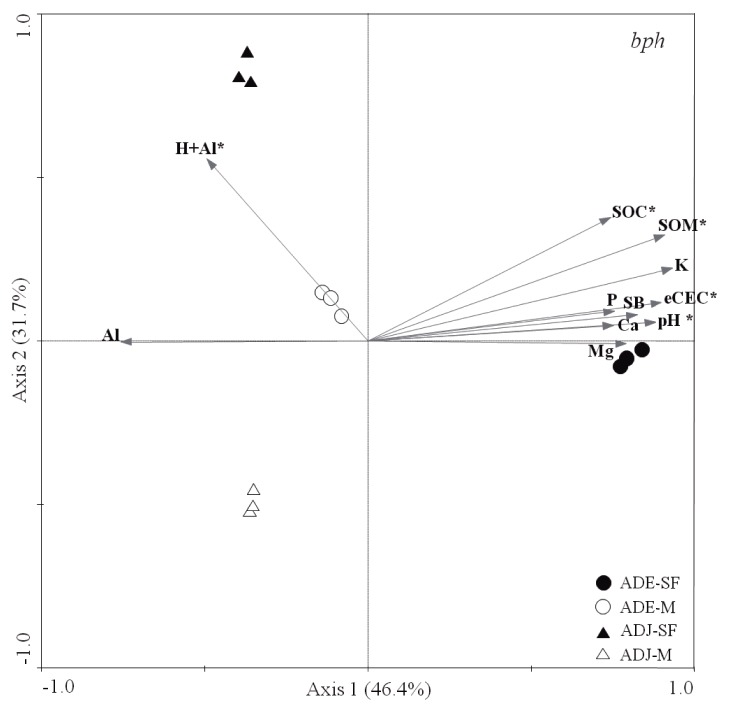
Redundancy analysis (RDA) based on T-RFLP data obtained for the *bph* gene, and soil properties, at the four studied sites. Arrows indicate correlation between the chemical parameters and community structure of samples. The significance of correlations was evaluated via Monte Carlo permutation test and it is indicated as follows: * *p*<0.05. ADE  =  Amazon Dark Earth; ADJ  =  Adjacent soils; SF  =  Secondary Forest; M =  Manioc cultivation.

Redundancy analysis based on T-RFLP data explained 78.1% of the variation in the first two axes, thus confirming the segregation of sites primary according to soil type, and further in relation to different land use types (Figure 3). Replicates within each soil site were very consistent, evidenced by the formation of concise clusters. We also observed that different soil types also correlated differently to measured chemical parameters. More precisely, the *bph* community structure from ADE-SF correlated mostly with pH, eCEC, SOM and SOC, while sites at ADJ-FS presented a significant correlation to H+Al.

ANOSIM analysis indicated statistical differences between the two soil types and land use systems (Table 1). R-values revealed a clear segregation of *bph* gene structures (R>0.75) in ADJ soils, while the 16S rRNA gene differed to a lower extent across sites (R<0.2).

**Table 1:** ANOSIM test for the bacterial 16S rRNA and *bph* gene based on T-RFLP data of the Amazonian Dark Earth (ADE) and Adjacent soil (ADJ) under secondary forest (SF) and under manioc cultivation (M).

	16S rRNA^a^		*bph* a	
	*R* values	p values	*R* values	p values
ADE × ADJ	0.17	<0.001	1.00	<0.001
ADE-SF × ADE-CULT	0.11	<0.001	1.00	<0.001
ADJ-SF × ADJ-CULT	0.03	<0.001	1.00	<0.001

a Samples were compared using T-RF peak height as a measure of abundance.

### Diversity of the bacterial *bph* gene across soil types and land uses

To access the composition of *bph* gene, samples were sequenced using 454-pyrosequencing. A total of 7,710 reads matched the barcodes, of which 6,877 reads passed the initial filtering (89.2%) and 5,965 (86.7%) were effectively translated into amino acid sequences using the FrameBot. A total of 4,690 valid amino acid sequences were further rarified to the depth of 750 sequences per sample (the minimum in a single sample) for comparative analysis.

The diversity indices for the *bph* gene (Table 2) revealed that ADE sites (SF and M) (*H*′ = 4.24 and 4.05, *L*′ = 0.024 and 0.028, respectively) were more diverse than ADJ sites (SF and M) (*H*′ = 3.19 and 3.17, *L*′ = 0.098 and 0.107, respectively). These indices also showed that sites under SF were more diverse than sites under M. Richness estimators (i.e. Chao1, ACE and Jackknife) also revealed ADE sites (SF and M) (Chao 1 = 238 and 229, ACE  = 248 and 286, Jackknife  = 271 and 255, respectively) to present higher values than ADJ (Chao 1 = 151 and 127, ACE  = 210 and 166, Jackknife  = 170 and 130, respectively), with SF sites being also higher than sites under M.

**Table 2:** Comparison of diversity indices and richness estimators for the *bph* gene.

Site	OPFs a	Richness			Diversity		ESC^ b^
		Chao 1	ACE	Jackknife	Shannon (H′)	Simpson (L′)	
ADE-SF	159	238	248	271	4.24	0.024	0,79
ADE-M	129	229	286	255	4.05	0.028	0,80
ADJ-SF	95	151	210	170	3.19	0.098	0,86
ADJ-M	90	127	166	130	3.17	0.107	0,87

a The operational protein family (OPFs), richness estimators (ACE, Chao1 and Jackknife), diversity indices (Shannon and Simpson) and ^b^estimated sample coverage were calculated at a cutoff value of 94% of sequence identity.

ADE  =  Amazon Dark Earth soils; ADJ  =  Adjacent soils; SF  =  Secondary forest; M =  Manioc cultivation.

ADE sites presented a total of 159 and 129 OPFs for SF and M sites, respectively. Conversely, these values for ADJ were lower (95 and 90, for SF and M, respectively). These sites also presented a different number of singletons (number of unique reads per OPF): 66, 57, 43 and 36 for ADE-SF, ADE-M, ADJ-SF and ADJ-M, respectively.

**Figure 4:**
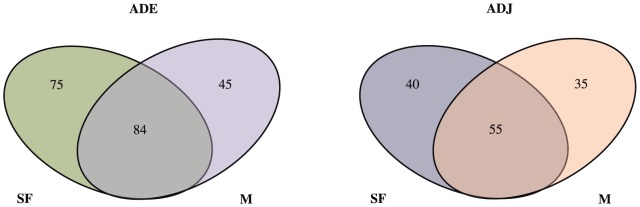
Venn's diagram of *bph* data belonging to operational protein families (OPFs) for different soil types under different land uses. ADE  =  Amazon Dark Earth; ADJ  =  Adjacent soils; SF  =  Secondary Forest; M =  Manioc cultivation. Sequences were grouped into OPFs based on sequence identity of 94%.

Statistical differences among sites for the composition of the *bph* gene were confirmed by ∫-Libshuff (*P*<0.001). Venn's diagrams highlight the number of shared OPFs among samples (Figure 4). The number of shared OPFs between ADE soils under different land uses systems (SF and M) was 84 (41% of the total OPFs presented in ADE sites). Conversely, the number of shared OPFs between ADJ soils under different land uses was 55 (42% of the total OPFs in ADJ sites). Soils under SF presented higher numbers of unique OPFs for both soils (ADE and ADJ) (75 and 40 unique OPFs for SF sites and 45 and 35 unique OPFs for ADE sites, respectively).

The estimation of Good's coverage revealed higher values for ADJ sites (0.86 for ADJ-SF and 0.87 for ADJ-M) than for ADE sites (0.79 for ADE-SF and 0.80 for ADE-M), suggesting a highest number of unique sequences in ADE sites. Rarefaction curves (Figure S1) indicate that ADE (SF and M sites) presented a more diverse community than ADJ (SF and M). Sites under SF (ADE and ADJ) were also comparatively more diverse than sites under M (ADE and ADJ). For all comparative analysis the sampling effort did not covered the richness of *bph* gene. The exception was observed for samples from ADJ sites, where a trend towards a “plateau” was observed.

**Figure 5:**
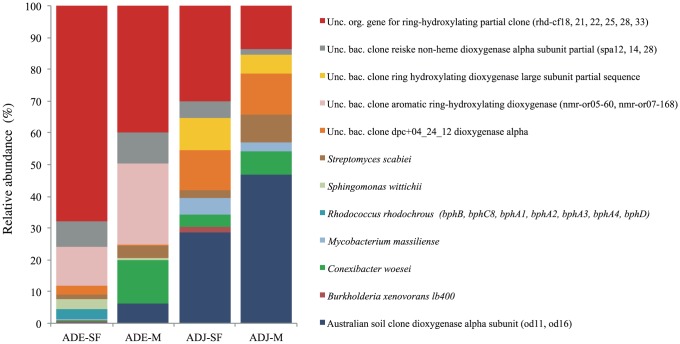
Bar charts representing the taxonomic affiliation of *bph* gene sequences. Sequences were affiliated using the TBlastX tool available in the GenBank database. ADE  =  Amazon Dark Earth; ADJ  =  Adjacent soils; SF  =  Secondary Forest; M =  Manioc cultivation.

The Principal Coordinate Analysis (PCoA) based on Unweighted UniFrac distances revealed distinct patterns in phylogenetic community composition (Figure 5). The first axis explained 59.59% of the data variation, and this axis separated samples according to soil type. The second axis explained 22.63% of the data variation, and this axis segregated samples according to land use system.

### Taxonomic composition of the *bph* gene

**Figure 6:**
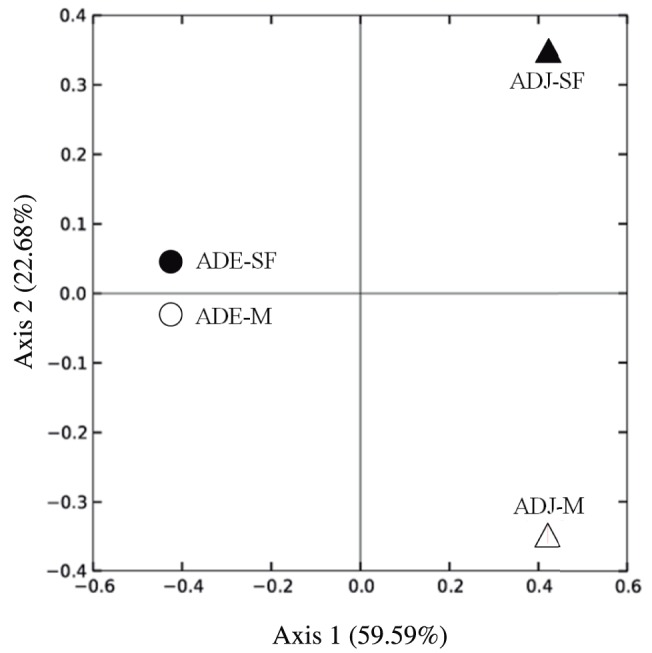
Principal Coordinate Analysis (PCoA) performed with the sequences obtained for the *bph* gene (based on Unweighted Unifrac distance). ADE  =  Amazon Dark Earth; ADJ  =  Adjacent soils; SF  =  Secondary Forest; M =  Manioc cultivation.

Each obtained OPF were further compared to sequences from the GenBank database for taxonomic assignment. All analyzed sequences matched translated proteins described as dioxygenases or putative dioxygenases, with E-values <10^−3^. The most abundant differences in dioxygenases (established as dioxygenases at least ten-fold higher in one site than another) are shown in Figure 6. Sequence matches were associated with aromatic hydrocarbon degradation genes belonging mostly to the genera *Streptomyces*, *Sphingomonas*, *Rhodococcus*, *Mycobacterium*, *Conexibacter* and *Burkholderia*, and uncultured bacterial clones. The taxonomic affiliation of the reads also revealed the predominance of the dioxygenase sequence belonging to unculturable organisms (rdh cf33) in ADE sites, and the predominance of the dioxygenase sequences similar to those previously described at Australian soil (od16) in ADJ sites.

### Quantification of the bacterial 16S rRNA and *bph* genes

**Figure 7:**
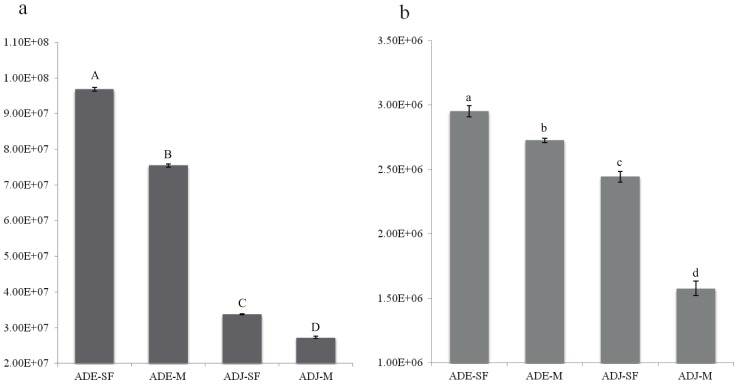
Log gene copy number (*y* axis) of (a) 16S rRNA and (b) *bph* genes across the studied sites. Sites are indicated in the *x* axis. Error bars represent the standard deviation of three independent replicates. ADE  =  Amazon Dark Earth; ADJ  =  Adjacent soils; SF  =  Secondary Forest; M =  Manioc cultivation. Different upper case letters refer to differences for the 16S rRNA across sites; while different lower case letters refer to differences for the *bph* gene (*P*<0.01, Tukey test).

Variations in bacterial 16S rRNA gene abundances were clear among the evaluated sites. Bacterial 16S rRNA gene ranged from 2.7×10^7^ (ADJ-M) up to 9.7×10^7^ (ADE-SF) copies per gram of soil. The results indicated that, soil type and land use influenced the abundance of bacteria in the analyzed samples, and higher values were found at ADE-SF site (Figure 7a).

The abundance of the *bph* gene ranged from 1.6×10^6^ (ADJ-M) to 2.9×10^6^ (ADE SF) copies per gram of soil. Conversely to the data obtained for the total bacteria (i.e. 16S rRNA gene data), the abundance of the *bph* gene was also higher at ADE-SF site (Figure 7b).

## Discussion

The aim of this study was to assess bacterial hydrocarbon degrading genes in anthropogenic sites from Brazilian Amazon comparatively to their adjacent locations, under two different land use systems. Recent studies have described the high fertility of ADE soils when compared to adjacent soils in the same area (ADJ), mostly because of their increased pH, higher cation exchange capacity, nutrient content and incompletely combusted biomass [3], [6], [8], [28], [38]. Although it is well-known that the taxonomic composition of bacterial communities is strongly influenced by pH [39], [40], the pH variation in our data indicate that this is also a strong predictor of the composition and diversity of the bacterial *bph* gene. Variations in soil pH, together with eCEC, SOC and SOM, collectively accounted for 78.1% of the total variation explained in the RDA plot based on T-RFLP data. Higher P values in ADE sites are likely to be an effect of pH, seeing that low acidity soils are known to increase the P solubility [28]. Also, the historical formation of ADE sites (constantly amended with bones and vegetation burning activities) is an intrinsic characteristic of this system, which could possibly explain the high content of phosphorus. There is also a direct relationship between soil P content and eCEC values in ADE sites mainly because the P adsorption decreases due to the formation of complex compounds between P and the organic matter present in the upper layer of the soil[41]. The values of eCEC in our samples were mostly correlated to the organic matter concentration, which was 2-fold higher in ADE than ADJ sites. These findings are likely explained by the higher amount of biochar found in these anthropogenic sites. Biochar is known for retaining soil nutrients due to its specific surface and negative charge density per unit of surface area [20], [42], [43]. In short, the collective chemical properties intrinsic of ADE sites may play an important role in their high levels of nutrient availability and, ultimately, their fertility. In this context, we hypothesize that such fertility has caused the ADE soils to harbor a higher microbial diversity and functionality than their adjacent soils. Agricultural practices can also alter soil properties, mostly by interfering on the biochemistry of the organic matter available in the system [44]. Our data revealed such an influence in the measurements of SOC, SOM and eCEC, which were significantly higher in ADE sites under secondary forest than under manioc cultivation system. Other chemical properties such as pH, SB and amount of P, Mg and H+Al, did not statistically differ between different land uses in ADE and ADJ sites.

Soil chemical data was also used in regression analyses to understand patterns in the microbial community structure of the *bph* gene. Microbial community structure can be defined by patterns of species abundance and population within a given community [45], which are mostly regulated by the ability of the microorganisms to interact among them and with local conditions [46]. In this study, T-RFLP results did not reveal significant differences among the richness of T-RFs between ADE and ADJ samples for the total bacterial community (16S rRNA). However, ANOSIM revealed significant differences in total bacterial community structure between ADJ and ADE soils types (R = 0.17) and between land uses (R = 0.11 for ADE-SF versus ADE-M; and R = 0.03 for ADJ-SF versus ADJ-M; Table 1). Lima [47], who similarly assessed the variation of ADE-SF, ADE-M, ADJ-SF and ADJ-M sites, and Cannavan [48], who investigated changes across different ADE sites, also found differences in 16S rRNA community structure between ADE and ADJ sites. Collectively, these studies support the idea that archaeological sites with a long history of anthropogenic activity influence the inhabiting microbiota of their respective soils.

Analysis of the community patterns for the bacterial *bph* gene revealed higher richness of T-RFs in ADE than in ADJ sites. Our results show that *bph* community changes are clear among sites (Figure 2), which can be observed by changes in the abundance of specific T-RFs across sites. In the same way, ANOSIM revealed significant differences in *bph* gene community structure between ADJ and ADE soils types (R = 1.00) and between land uses (R = 1.00 for ADE-SF versus ADE-M; and R = 1.00 for ADJ-SF versus ADJ-M; Table 1). We suspect, despite all variables present in our system, that the high presence of biochar, which chemically is formed by an aromatic polycyclic structure, could be a factor influencing the observed differences. Táncsics et al [49], by analyzing the structure community of catechol 2,3-dioxygenase genes in aromatic hydrocarbon contaminated environments by T-RFLP, observed that T-RFLP chromatograms obtained from contaminated samples had entirely different T-RFs compared to the control non-contaminated sample.

Redundancy analysis revealed *bph* gene structure to be correlated with different soil parameters (Figure 4). Briefly, ADE-SF correlated with pH, eCEC, SOM and SOC. ADJ-SF showed a significant correlation with H+Al. Several studies have suggested that variations in soil pH and properties related to soil acidity (e.g., K, Al, base saturation) are stronger predictors of the richness and diversity of inhabiting microbial communities [6], [18], [50], [51], [52], [53]. We extend this concept by advocating that other factors also might play a role in structuring the *bph* gene communities. For instance, the quantity and/or quality of soil organic matter (SOM) and its fractions are likely to regulate microbial community composition and associate function [54]. Since hydrocarbon degradation is one step into the carbon cycle, *bph* degraders might have an advantage in ADE sites by harnessing energy from elevated levels of SOC and SOM.

Quantification of total bacterial (16S rRNA) and *bph* gene abundances (Figure 7) revealed higher copy numbers of these genes in ADE sites, peaking at ADE-SF. These results indicate that soil characteristics of ADE, as well as their land uses has an influence on the abundance of both analyzed genes. Ding et al [55] analyzed the abundance of total bacteria using the16S rRNA gene and the abundance of polycyclic aromatic hydrocarbons ring-hydroxylating dioxygenase (PAH-RHD_α_) genes by qPCR in two different phenanthrene-contaminated soils (i.e. Luvisol and Cambisol). Their results revealed a significantly higher 16S rRNA gene copy number per gram of soil in both phenanthrene-contaminated soils compared to their controls. PAH-RHD genes were detected only in contaminated soil samples, and the values ranged from 2.0×10^7^ (Luvisol phenanthrene-contaminated soil) to 1.7×10^6^ (Cambisol phenanthrene-contaminated soil) copies per gram of soil, showing similar values to those found in our study. Similarly, a study about the diversity of naphthalene dioxygenase genes (*nahAc*) in soil environments from the Maritime Antarctic revealed that the quantities detected in bulk and rhizospheric soils from PAH-affected sites ranged from 6.4×10^4^ to 1.7×10^6^ *nahAc* gene copies per gram of soil and presented significantly higher abundance compared with the corresponding counterparts of bulk and rhizospheric soils from non-polluted sites [56].

We used 454-pyrosequencing to investigate the community composition of the *bph* gene. The use of a high-throughput culture-independent approach enabled an overview of the taxonomic groups occurring in the sampled sites. Taxonomic analyses of 3,000 partial sequences of the *bph* gene revealed that this gene was most represented by bacteria belonging to the genera *Streptomyces, Sphingomonas, Rhodococcus, Mycobacterium, Conexibacter* and *Burkholderia*, in addition to sequences matching uncultured bacteria (Figure 5). Overall, these groups encompass mixed sequences of toluene/biphenyl dioxygenases, such as *bph*B, *bph*C8, *bph*A1, *bph*A2, *bph*A3, *bph*A4 and *bph*D, and enzymes encoding the alpha subunit of dioxygenases for the degradation of Polycyclic Aromatic Hydrocarbons (PAHs), which are described as environmental widely distributed dioxygenases. Other studies targeting dioxygenases have also reported sequences belonging to *Sphingomonas*, *Rhodococcus, Mycobacterium, Conexibacter* and *Burkholderia* as major taxonomic groups [21], [28], [57]. Zhou et al [58] were also able to isolate aromatic hydrocarbon degrading bacteria from mangrove sediments, which were classified as the genera *Mycobacterium* and *Sphingomonas*. Members of these genera have commonly been isolated from diverse sediments and soils [59], [60], and they play an important role in hydrocarbon biodegradation [61], [62], [63]. For instance, Ding et al [55] who studied the diversity of dioxygenases using clone libraries for aromatic ring hydroxylating dioxygenases (ARHD) genes identified gene sequences corresponding to the *phnAc* gene belonging to the *Burkholderia* sp. strain Eh1-1 and PAH-RHD genes of the *Mycobacterium* sp. strain JLS.

Confirming the results of T-RFLP analysis, differences among *bph* communities for ADE and ADJ sites and between SF and M land uses were also observed by differences in their Shannon and Simpson diversity indices (Table 2). Similarly, Germano et al [28] reported a higher diversity of ARHD genes in ADE sites under secondary forest rather than under agricultural cultivation, suggesting that deforestation in these sites has an influence on the diversity of these catabolic genes. We observed that in both soil types, the number of OPF (Table 2) was higher in SF samples, also indicating an influence of the land use system on the richness of this gene. According to Jesus et al [51], the land conversion of tropical forest to agricultural use modifies the size, activity and composition of soil microbial communities. This deeply influences specific bacterial functions, including those acting on organic matter decomposition and nutrient cycling in soils.

The intensive land use by agricultural practices and the conversion of Amazon soil into agricultural areas has been reported to cause significant shifts in the chemical properties of the soil, such as variations in SOC, SOM and eCEC, leading towards a homogenization of the inhabiting bacterial community [64]. However, we hypothesized that, despite an effect of land use, greater differences in community composition would be observed according to soil type. We also expected communities in these different soils to respond differently to agricultural practices. In this context, the literature describes the resilience phenomenon as the ability of the soil to cope with external disturbances and to retain its functional capacity upon the imposition of a stress [65], [66], [67]. We observed that higher differences in the diversity of *bph* occurred between the different land uses in ADJ sites rather than in ADE sites. Thus, land use type appeared to have a stronger effect on the *bph* community in ADJ sites, maybe due to the higher resilience of the ADE soil against agricultural practices. Principal Coordinate Analysis (PCoA) performed for the *bph* gene also supports these results (Figure 6), revealing that ADE sites (SF and M) clustered closer to each other than observed for ADJ sites (SF and M).

The taxonomic analyses of sequences also revealed that ADE sites harbored distinct *bph* phylogenetic structure from ADJ. These results suggest that the heterogeneity of bacterial *bph* communities could be related to their ability to respond to differences of land use type and soil chemical properties. In a previous study Germano et al. [28] compared the phylogenetic structure of *bph* sequences from ADE sites and revealed that most of the protein clusters from these sites group apart from the main well-known dioxygenase groups previously proposed by Kweon et al [27].

In conclusion, we have taken a distinct and highly diverse soil to elucidate the ecological properties and taxonomic affiliation of bacterial communities characterized by the presence of the *bph* gene, which is crucial to the biodegradation of aromatic compounds. These results enable us to understand differences in the structure, abundance and composition of the main active organisms in ADE soils when compared to their adjacent locations. Further studies focusing on the catabolic activities of these communities are needed to enable a collective view of the formation, dynamics and maintenance of functional properties in ADE soils.

## Supporting Information

Figure S1 Rarefaction curves of bacterial *bph* gene sequences were grouped into OPF based on distance sequence of 0.06. ADE  =  Amazonian Dark Earth soils; ADJ  =  Adjacent soils; SF  =  Secondary forest; M =  Manioc Cultivation.
(EPS)

Table S1 Soil chemical properties of the studied sites. Amazonian Dark Earth (ADE) and Adjacent soil (ADJ). Sites were located under two different land uses: Secondary forest (SF) and under Manioc (*Manihot esculenta*) cultivation (M). Significant differences between sites are followed by different letters (*P*<0.05, Tukey test).
(DOCX)
