# Supplementary material for: Assessment of Bacterial bph Gene in Amazonian Dark Earth and Their Adjacent Soils
Source: PLoS One. 2014 Jun 13;9(6):e99597. doi: 10.1371/journal.pone.0099597 (PMC4057261; doi:10.1371/journal.pone.0099597)
Supplement: Table S1 — Soil chemical properties of the studied sites. Amazonian Dark Earth (ADE) and Adjacent soil (ADJ). Sites were located under two different land uses: Secondary forest (SF) and under Manioc (Manihot esculenta) cultivation (M). Significant differences between sites are followed by different letters (P<0.05, Tukey test). (DOCX) [file pone.0099597.s002.docx]

**Supporting information**

**Tables**

Table S1. Soil chemical properties of the studied sites. Amazonian Dark Earth (ADE) and Adjacent soil (ADJ)^1^. Sites were located under two different land uses: Secondary forest (SF) and under Manioc (*Manihot esculenta*) cultivation (M). Significant differences between sites are followed by different letters (*P* < 0.05, Tukey test).

| Chemical properties^(2)^ | ADE-SF | ADE-M | ADJ-SF | ADJ-M |
| --- | --- | --- | --- | --- |
| pH in H_2_O | 5.51^a^ | 5.41^a^ | 3.53^b^ | 3.74^b^ |
| SOC (g kg^-1^) | 38.96^a^ | 28.23^b^ | 20.99^c^ | 16.16^c^ |
| SOM (g kg^-1^) | 67.02^a^ | 48.55^b^ | 31.7^c^ | 27.79^c^ |
| P (mg dm^-3^) | 143^a^ | 144^a^ | 9^b^ | 4^b^ |
| K (mg dm^-3^) | 70^a^ | 62^b^ | 35^b^ | 25^b^ |
| Ca (cmol_c_ dm^-3^) | 10.41^a^ | 7.77^b^ | 0.64^c^ | 0.44^c^ |
| Mg (cmol_c_ dm^-3^) | 1.43^a^ | 1.33^a^ | 0.25^b^ | 0.45^b^ |
| Al (cmol_c_ dm^-3^) | 0.03^a^ | 0.03^a^ | 2.06^b^ | 1.77^c^ |
| H+Al (cmol_c_ dm^-3^) | 8.46^a^ | 7.85^a^ | 13.07^b^ | 13.23^b^ |
| SB (cmol_c_ dm^-3^) | 12.9^a^ | 10.33^a^ | 1.14^b^ | 0.64^b^ |
| eCEC (cmol_c_ dm^-3^) | 13^a^ | 9.09^b^ | 3.2^c^ | 2.17^c^ |

^(1)^Means (n = 3) from each sampling point, in each site.

^(2)^Abbreviations: SOC, soil organic carbon; SOM, soil organic matter; SB, sum of the bases (Ca+Mg+K), eCEC, effective cation exchange capacity.
